# Simulating drone and bodily movements: a behavioral study

**DOI:** 10.3389/fpsyg.2025.1559756

**Published:** 2025-04-25

**Authors:** Anna Kolesnikov, Marta Calbi, Martina Montalti, Nunzio Langiulli, Michele Guerra, Vittorio Gallese, Maria Alessandra Umiltà

**Affiliations:** ^1^Department of Art History, Film and Audiovisual Media Studies, Université de Montréal, Montreal, QC, Canada; ^2^Unit of Neuroscience, Department of Medicine and Surgery, University of Parma, Parma, Italy; ^3^Department of Food and Drug, University of Parma, Parma, Italy; ^4^Department of Ancient and Modern Civilizations, Polo Universitario “Annunziata”, University of Messina, Messina, Italy; ^5^Department of Humanities, Social Sciences and Cultural Industries, University of Parma, Parma, Italy; ^6^Italian Academy for Advanced Studies in America, Columbia University, New York, NY, United States

**Keywords:** embodied simulation, ascent, movement, drones, naturalistic video stimuli

## Abstract

**Introduction:**

This study explores how drone movements and human bodily gestures influence spectators’ perceptions, focusing on physical and emotional involvement, aesthetic appreciation, and time perception.

**Methods:**

Inspired by the iconic staircase scene from the Soviet film The Cranes Are Flying (1957), a set of 81 naturalistic video stimuli was created using a drone-mounted camera, varying in Drone Movement (Ascending, Descending, Still), Human Presence (Female, Male, None), and Image Speed (Normal, Low, Very Slow). Participants evaluated each video based on Liking, Perceived Movement, Physical Involvement, Emotional Involvement and Perceived Duration.

**Results and discussion:**

Results showed that ascending movements elicited the highest levels of perceived movement, aesthetic appreciation and emotional engagement, outperforming descending and still movements. These results could be explained by a stronger sense of effort and exertion associated with ascending movements, aligning with the embodied simulation of upward motion against gravity. Human presence significantly enhanced ratings across all metrics compared to videos without human figures, thus suggesting that bodily movements play a crucial role in evoking stronger viewer involvement. Additionally, the Female condition received higher aesthetic ratings. Notably, normal image speed yielded greater perceived movement and physical involvement than slowed footage, highlighting a stronger connection to the natural rhythm of bodies in motion. Furthermore, ascending and descending conditions were perceived as lasting longer than still, corroborating prior research on time perception distortions with dynamic stimuli. Correlation analysis highlighted a strong link between physical involvement, emotional engagement, and aesthetic appreciation, underscoring the interplay between bodily and emotional responses. This study emphasizes the potential of drone-based cinematography to evoke embodied and emotional responses, reinforcing the role of embodied simulation theory in cinematic experiences.

## Introduction

According to embodied simulation theory, humans tacitly “simulate” the actions of the other by mapping them in the sensorimotor cortex of their brain (Gallese, 2005; Gallese and Sinigaglia, 2011; Gallese, 2014). Indeed, a growing body of evidence supports the existence of a link between action execution and perception in humans, which forms the foundation of social cognition. Moreover, the observed context sensitivity of visuomotor and sensorimotor activation supports the idea that these motor simulation processes are finely tuned to facilitate specific social interactions (e.g., Babiloni et al., 2002; Muthukumaraswamy et al., 2004; Calvo-Merino et al., 2005, 2006; Orgs et al., 2008; Streltsova et al., 2010; Abreu et al., 2012; See also Gallese, 2009; Bonini et al., 2022).

This direct link between perception and action has been also contributing a contemporary, interdisciplinary reassessment of how the brain–body system is engaged during the film experience, forming the theoretical framework of “embodied cinema” (Carluccio and Villa, 2006; Eugeni and D’Aloia, 2014; Tikka and Kaipainen, 2014; Coëgnarts and Kravanja, 2015; Eugeni, 2018; Gallese and Guerra, 2012, 2020). According to this framework, the meaning-making process in film is considered to be inextricably linked to the interrelation between the brain, body and environment of the viewer (Gallese and Guerra, 2012, 2020). According to Gallese and Guerra, embodied simulation theory can enrich film studies at the receptive and creative levels, shedding new light on at least three types of “film embodiment”: (1) acting style, (2) film style, and (3) and the spectator’s responses to filmed bodies and objects (Gallese and Guerra, 2012). The first stage of embodiment, i.e., acting, which brings the audience into the forefront of “action” and “tactility,” and film style (e.g., camera movements), emerge as a “negotiation” with the acting body. The role of the camera is integral, endowing the cinematic experience with kinesthetic and tactile cues that animate the film with “vitalizing” qualities and a subjectivity of its own (Gallese and Guerra, 2014).

In support of this, recent research has demonstrated the role of camera movements in evoking sensorimotor resonance in viewers. Heimann et al. (2014) showed that the Steadicam elicits a stronger senorimotor resonance compared to a “zoom.” This was explained by the greater sense of “being there” that the Steadicam affords. Movie clips filmed with the Steadicam were indeed rated by participants as more engaging, natural and closest to the actions of an approaching observer. The stronger motor resonance measured in the Steadicam condition may also be driven by motor engagement with the “trace” of the Steadicam’s own movement across the scenic space. Replicating the study in an empty room, Heimann et al. (2019) found that greater motor resonance was again evoked for the Steadicam, providing the first empirical evidence that camera movement alone can modulate spectator’s bodily engagement during film experience. Drawing on the studies, Yilmaz et al. (2023) investigated the relationship between camera movement techniques and audience cognitive responses. Their behavioral study explored how different camera movement methods affect viewers’ immersion and emotional responses to dramatized scenes with a particular focus on enhancing the ecological validity of the stimuli.

Another aspect that has drawn the attention of the embodied cinema framework, is the impact of motion-related properties of visual stimuli on spectators’ experience of time (e.g., Liapi et al., 2024; see also the embodied account of time perception: Droit-Volet et al., 2013, 2020; Wittmann, 2014). Research has indeed shown that moving stimuli are perceived as lasting longer than stationary ones, even if their actual duration is the same. Studies have also demonstrated that speed and temporal frequency can alter viewers’ perception of time (e.g., Brown, 1995; Kanai et al., 2006; Eagleman, 2008). In this vein, Balzarotti et al. (2021) recently examined whether the cinematographic editing density affects viewers’ perception of time showing that participants overestimated the duration of fast-paced videos compared to the master-shots.

In light of the previous evidence on the role of camera movement in evoking an embodied response in the spectator, the question arises naturally: could camera-mounted drones be considered as *flying Steadicams*? Drone models utilize gimbal suspensions, which stabilize the camera and prevent vibration, similar to Steadicams. In fact, a drone’s visual imagery goes beyond “mere aerial photography” (Ledet Christiansen, 2020, p. 286), resembling that of body-mounted cameras, allowing drones to imitate Steadicams. Just as the Steadicam enabled previously impossible shots, drones offer previously impossible points of view (POVs). The “non-human” floating sensorium of drone vision creates a remote, “unmanned” presence that both enables and “challenges” embodiment. Going beyond being an “extension of man,” as per McLuhan’s (2001) dictum, the “multisensory mobilities” of camera-mounted drones push human vision beyond the limits of embodiment, into a previously “unoccupiable” sensorium. Here, the once “anthropomorphic” qualities of camera movement (Bordwell, 1977) take on a different mode of being. Although there is a growing number of theoretical (Virilio, 1994; Verhoeff, 2012; Campbell, 2018; Agostinho et al., 2020; Ledet Christiansen, 2020; Jablonowski, 2020) and technological (Eriksson et al., 2019; Cherpillod et al., 2019) studies on the sensorimotor capacities of drone flight, so much so as to define it as an embodied “technology of mediation” (Agostinho et al., 2020; Garrett and McCosker, 2017), to date, no studies have investigated the effect of drone footage with and without human bodily movement on spectators’ cognitive behavioral mechanisms.

To further test the predictions of the framework of embodied cinema and investigate the embodied responses to human and camera movement in aerial shots, we recently developed an original experimental project. In line with the growing trend of using naturalistic stimuli for cognitive science research on audiovisual media (Hasson et al., 2010; Sonkusare et al., 2019; Jääskeläinen et al., 2021; Saarimäki, 2021; Tikka et al., 2023), we created a novel, ecologically valid set of video stimuli filmed by means of a drone. Specifically, as a novel contribution to experimental methods, the stairway scene from the award-winning Soviet film The Cranes Are Flying (Kalatozov, 1957) was chosen as aesthetic model. This selection allowed for the construction of highly controlled video clips, with careful attention to relevant variables, while simultaneously maintaining a high standard of artistic quality (for details see Kolesnikov, 2022). These stimuli mimic real-world situations and, unlike the static visual stimuli commonly used in experimental studies, they present dynamic, immersive camera movements in a more naturalistic and complex context. The clips were developed in collaboration with a cinematographer and drone pilot, targeting perception of motion. One female and one male actor were instructed to run up and down a staircase while the drone tracked their movements, ascending and descending the stairwell vertically. For the control condition, both the actors and the drone remained still. The drone also filmed ascending, descending, and still variants without the presence of the actors (see the “Materials and Methods” section below for more details). In a parallel study, audio stimuli in corresponding variants were recorded, demonstrating that participants linked ascending musical movement with increased effort or exertion, leading to a heightened emotional response with respect to descending and flat conditions (Kolesnikov, 2022; Kolesnikov et al., 2023).

Thus, the aims of the present study are to validate the film clips, presented without sound, investigating the impact of Drone Movement (Ascending, Descending, Still), Human Presence (Female, Male, None [no human]) and Image Speed (Normal, Slow, Very Slow) with respect to spectators’ ratings of Liking, Perceived Movement, Physical Involvement, Emotional Involvement, and Perceived Duration. We hypothesize that: (1) Female and Male Human Presence will be perceived as evoking significantly greater Movement, Physical Involvement, Emotional Involvement and Liking with respect to None (i.e., No Human Presence); (2) Ascending Drone Movement will be perceived as evoking greater Movement, Physical Involvement and Emotional Involvement than Descending and Still (due to greater perceived effort/exertion); and (3) Very Slow and Slow Image Speed will be perceived as evoking significantly longer Duration, and greater Liking, Movement, Emotional Involvement and Physical Involvement with respect to Normal (due to greater perceived effort/exertion).

## Materials and methods

### Participants

Participants were recruited through opportunity sampling using Facebook, which filtered individuals for age and residence in Parma (Italy). Interested participants were further screened using a survey, and individuals with professional filmmaking or film studies backgrounds were excluded from the study. In total, 31 healthy volunteers of Italian nationality took part in the experiment: 14 female and 17 males, mean age 25.03 (Standard Deviation – SD = 4.63, min = 18, max = 35). All participants reported having normal or corrected-to-normal visual acuity. All participants were either right-handed or ambidextrous (as determined by the Edinburgh Handedness Inventory; Oldfield, 1971). Power was calculated a posteriori by means of G*Power 3.1 (Faul et al., 2007) using the linear multiple regression: random model to test for a linear mixed effect model for each dependent variable. With a H1 *ρ* equal to 0.6 (large effect size), an alpha level of 0.05, 3 predictors, and a total sample size of 31 resulted in an actual power of >0.9. All participants provided written informed consent to participate in the study, which was conducted in accordance with the Declaration of Helsinki (2013), complying with the Ethical Code for Psychological Research of the Italian Psychological Society and the Ethical Committee of the Area Vasta Emilia Nord (AVEN. REF: 85/2019/DISP/UNIPR).

### Stimuli

**Figure 1:**
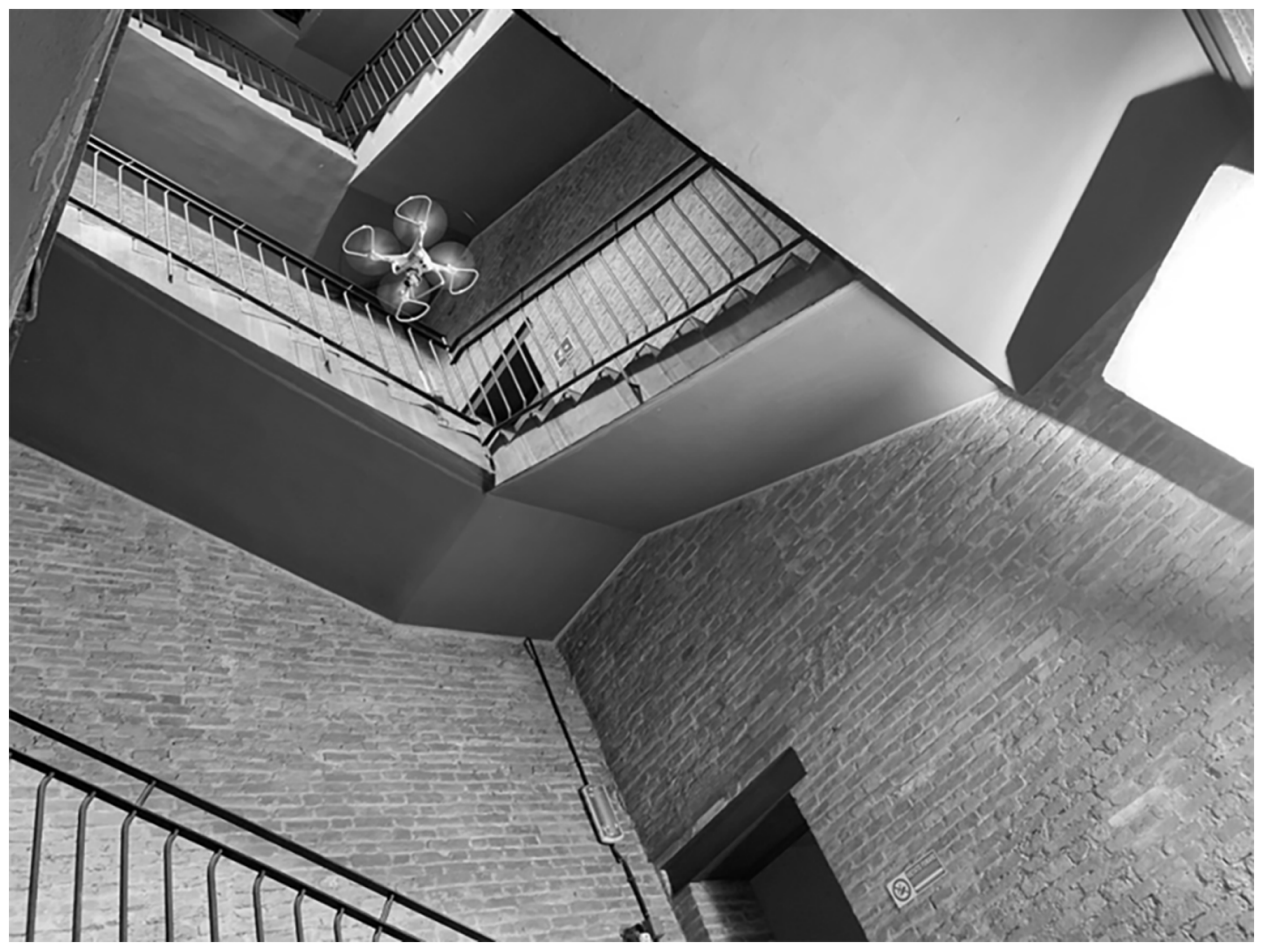
Production. Shooting the videos using a DJI Phantom 4 Pro Drone.

**Figure 2:**
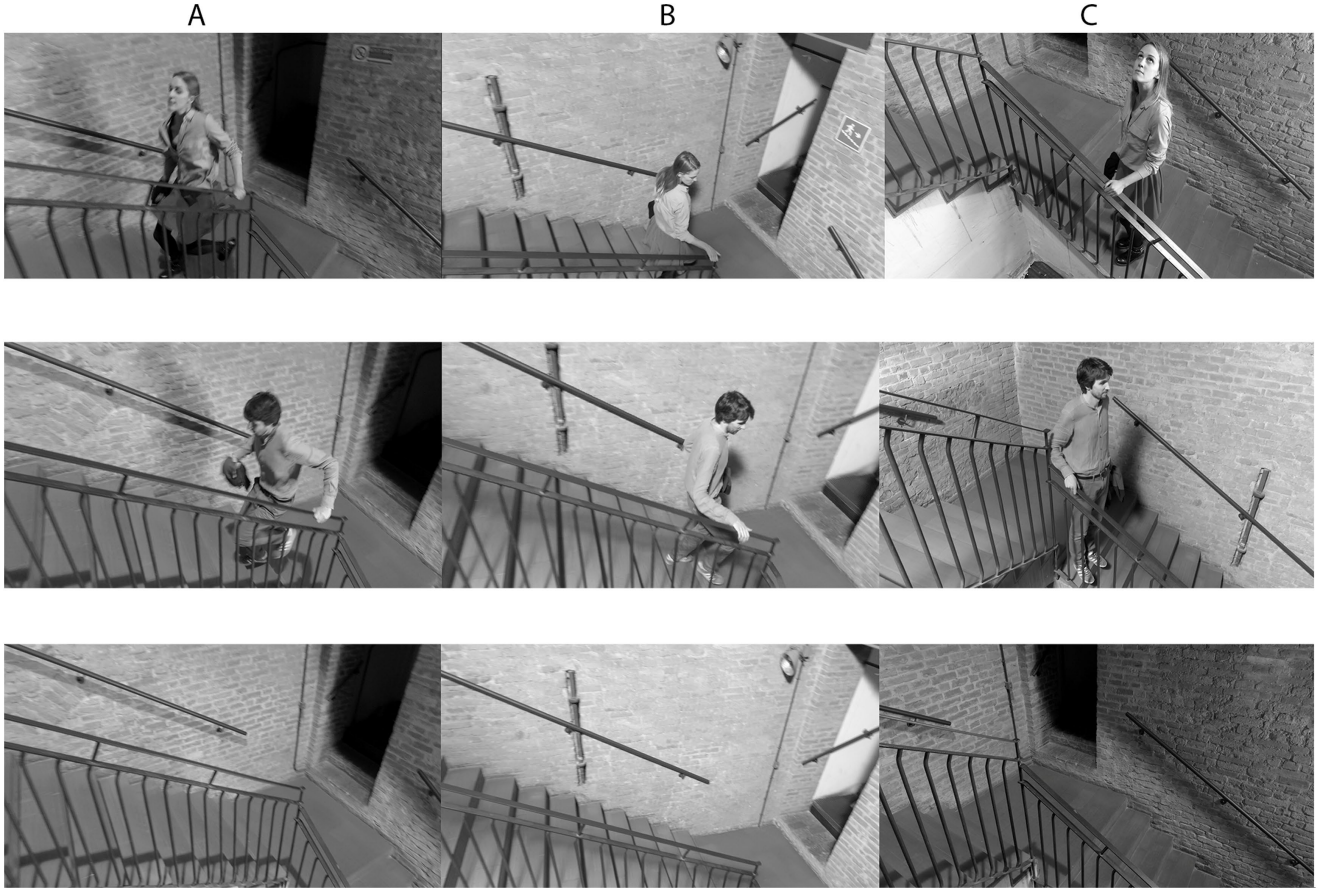
Stimuli. Ascending conditions for Female, Male and None, respectively **(A)**; Descending conditions for Female, Male and None, respectively **(B)** and Still conditions for Female, Male and None, respectively **(C)**.

The stimuli were modeled after the staircase scene from the film *The Cranes Are Flying* (Kalatozov, 1957). In the film, an innovative “elevator crane” was constructed, featuring a cradle for the cinematographer and his camera to execute the technically demanding scene, which was pulled along iron poles with circular operator rails to achieve a seamless lateral tracking shot while ascending the stairwell (see Kolesnikov, 2022). In the present study, a drone was employed to replicate the intricate, circular motion of the scene’s complex camerawork, avoiding the time-consuming and costly task of constructing an elevator crane or levy. Specifically, the videos were filmed with a DJI Phantom 4 Pro Drone. The field of view of the camera installed on the DJI Phantom 4 PRO is: FOV 84° 8.8 mm/24 mm (35 mm format equivalent). One female and one male actor were instructed to run up and down a staircase while the drone completed an aerial shot on a vertical axis, ascending and descending the stairwell vertically. For the control condition, both the actors and the drone remained still (see Figure 1). The gimbal mode of the drone was configured to its default settings, i.e., “Follow” mode, where the gimbal is automatically adjusted to maintain a level horizon line to ensure a stabilized image. The commands sent by the pilot were: (1) yaw (rotation on its vertical axis), and (2) throttle (increase/decrease in altitude). This combination enabled the drone to follow the moving actors with precision. The maintenance of the position on the point of ascent was ensured by the optical sensors that point to the floor, given a total absence of GPS connection. Due to poor lighting, a texture was applied on the floor in order provide references for the sensors. The drone also filmed ascending and descending variants without the presence of the actors. During filming, the drone maintained a constant distance from the actors (approximately 2 m) while it rotated on its axis. For the control condition, both the actors and the drone remained still. Premiere Pro CC was used to edit the raw drone footage into experimental stimuli in MP4 format with H.264 codec and a resolution of 1920 × 1,080 pixels (see Figure 2). Each clip had a frame rate of 25 frames per second, with a total of 250 frames, or 10,000 ms per clip. A cross dissolve of 25 frames (1,000 ms) was included at the beginning and end of each clip to create a more fluid transition between the fixation cross and stimulus frames. To control for possible confounding effects, clips were grayscaled.

Video clips were created combining the following factors and their levels: Drone Movement (Ascending, Descending, Still), and Human Presence (Female, Male, None), resulting in 27 clips These 27 clips with Image Speed Normal (100%) (250 frames/clip), were then also slowed down to 75% of the original image speed (Image Speed: Slow, 333 frames/clip), and to 50% of the original image speed (Image Speed: Very Slow, 500 frames/clip), for a total of 81 experimental video stimuli (see also Table 1). In order to ensure that there were no relevant significant differences across conditions with respect to motion and luminance, control analyses were performed on these parameters (see Supplementary material). All clips can be viewed at https://osf.io/d6wzt/?view_only=f2ba6e7164fe4d36bb24ff5ee457d51f.

**Table 1:** Stimuli/conditions.

Stimulus number	Drone movement	Human presence	Image speed
1–27	Ascending	Female	N,S,VS
		Male	N,S,VS
		None	N,S,VS
28–54	Descending	Female	N,S,VS
		Male	N,S,VS
		None	N,S,VS
55–81	Still	Female	N,S,VS
		Male	N,S,VS
		None	N,S,VS

N, normal; S, slow; VS, very slow.

### Procedure

Upon arrival, participants were asked to make themselves comfortable and were given instructions about the study. The experimental session consisted of two different and randomized phases.

In the first phase, participants were asked to fill out a series of questionnaires. The Interpersonal Reactivity Index (IRI; Davis, 1983) was used to measure empathy as a multidimensional construct. Motor imagination was assessed in all participants using the Vividness of Movement Imagery Questionnaire-2 (VMIQ-2; Roberts et al., 2008) with three subscales: External Visual Imagery (i.e., imagining yourself carry out the movement as you observe from the outside), Internal Visual Imagery (i.e., imagining yourself carry out the movement through your own eyes), and Kinesthetic Imagery (i.e., imagining the physical sensation of carrying out the movement). Immersive tendencies in different mediated environments were assessed using the Immersive Tendency Questionnaire (ITQ; Witmer and Singer, 1998).

**Figure 3:**
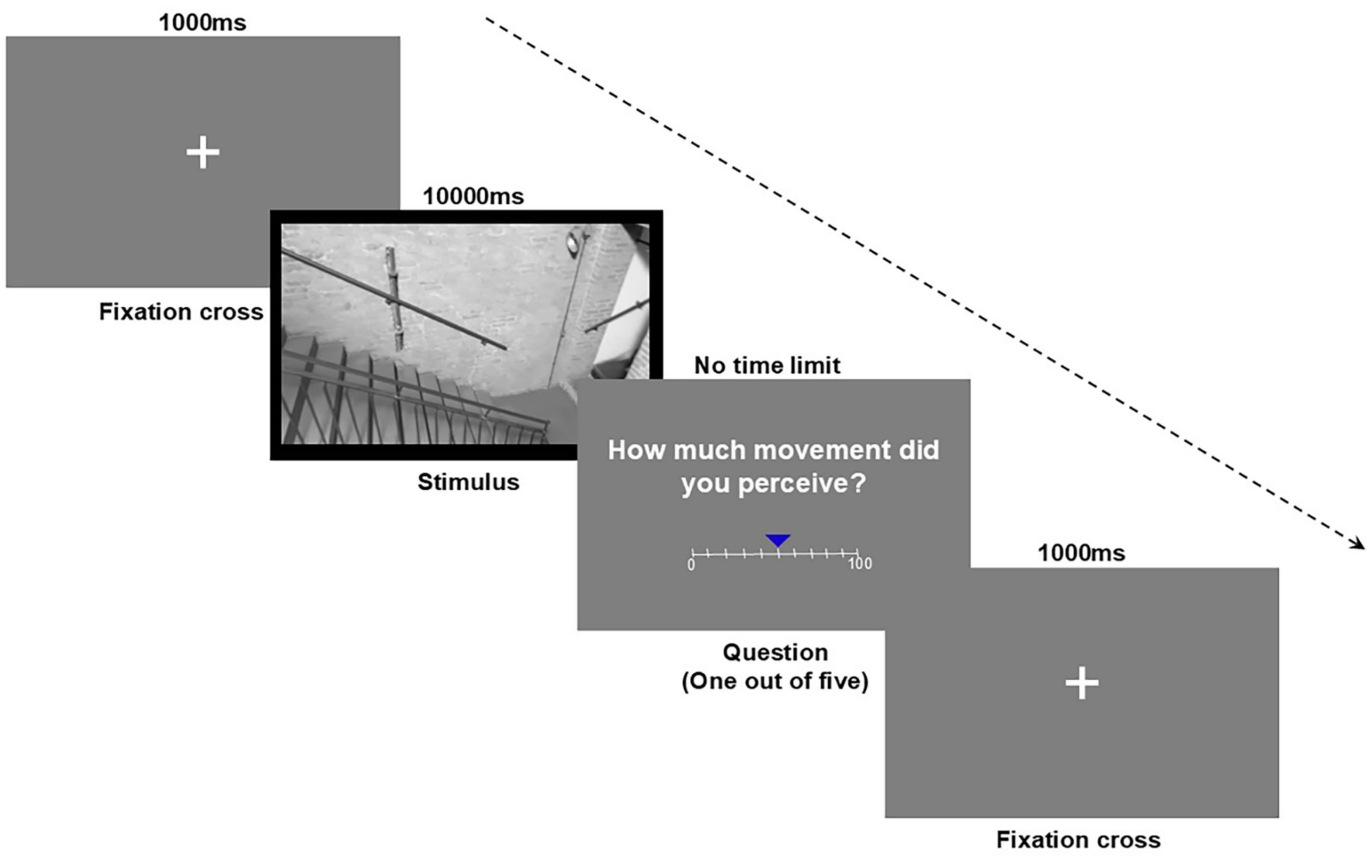
Example of experimental trial. Components: fixation cross frame (1,000 ms), stimulus frame (10,000 ms) and the Visual Analogue Scale (VAS) rating task (scale from 0 to 100, no time limit).

In the second phase, participants were asked to perform a computer task in which the 81 video stimuli were presented in randomized order. In each trial, a white fixation cross was presented on a gray background for 1,000 ms, a video stimulus was presented for 10,000 ms, and one question randomly selected from a pool of five questions was presented with no response time limit (Figure 3). The questions were: (1) “How much did you like it? (Liking); (2); “How much movement did you perceive?” (Perceived Movement); (3) “How physically involved did you feel?” (Physical Involvement); (4) “How emotionally involved did you feel?” (Emotional Involvement); and (5) “How long was the duration of the stimulus?” (Perceived Duration). Participants were asked to answer the questions as quickly and as accurately as possible, using the mouse, on a Visual Analogue Scale (VAS) ranging from 0 (very little) to 100 (very much). Each stimulus was repeated 5 times, followed each time by one of the 5 questions, for a total of 405 trials.

Before carrying out the experimental procedure, participants were presented with a brief training phase to become accustomed with the task. After the experimental session participants were asked to fill out a short debriefing survey about their experience. The experimental session was conducted in a quiet room, on a screen positioned approximately 60 cm from the participant. The experimental task was programmed using Psychopy 3.0 software (Peirce et al., 2019).

### Statistical analysis

To investigate whether VAS ratings given by paticipants were modulated by Drone Movement, Human Presence and Image Speed, a linear mixed effect analysis was carried out. Separately for each question (Liking, Perceived Movement, Physical Involvement, Emotional Involvement, and Perceived Duration), participants’ ratings were entered as dependent variables, (Drone Movement) (3 levels: Ascending, Descending, Still), Human Presence (3 levels: Female, Male, None) and Image Speed (3 levels: Normal, Slow, Very Slow) as independent fixed variables, and participant intercepts as random effects. Tukey’s test was used for post-hoc comparisons among means. Moreover, non-parametric Spearman rank correlations were performend among participants’ ratings to the five four questions. The critical probability value was corrected for multiple comparisons using the Bonferroni method (*p* = 0.05/10 = 0.005). To ensure there were no significant differences related to participants’ gender, control analyses were performed using *t*-tests (see Supplementary material). All analyses were performed using R software (R Core Team, 2019) and lme4 (Bates et al., 2015), effects (Fox, 2003) and emmeans (Lenth, 2022) packages; for data visualization, the ggplot2 package was used (Wickham, 2016).

## Results

### Liking

The model explained 63.62% of the variance in Liking ratings, taking into account the random effects (*R*^2^_m_ = 0.06, *R*^2^_c_ = 0.64). The model revealed a significant main effect of Drone Movement [*χ*^2^_(2)_ = 29.37, *p* < 0.001], showing that participants liked Ascending more than Descending [*t*_(779)_ = 2.7 *p* < 0.05], Ascending more than Still [*t*_(779)_ = 5.41, *p* < 0.001], and Descending more than Still [*t*_(779)_ = 2.72, *p* < 0.05]. A significant main effect for Human Presence was found [*χ*^2^_(2)_ = 72.26, *p* < 0.001], showing that participants liked Female more than Male [*t*_(779)_ = 3.58, *p* < 0.01], Female more than None [*t*_(779)_ = 8.47, *p* < 0.001], and Male more than None [*t*_(779)_ = 4.89, *p* < 0.001]. A significant main effect for Image Speed was found [*χ*^2^_(2)_ = 14.6, *p* < 0.001], showing that participants liked Normal more than Slow [*t*_(779)_ = 3.17, *p* < 0.01], and Normal more than Very Slow [*t*_(779)_ = 3.43, *p* < 0.01]. Interactions effects were not significant. See Table 2 for descriptive statistics.

**Table 2:** Estimated marginal means (M) and standard errors (SE) of fixed effects for each question.

	Drone movement			Human presence			Image speed		
	Ascending	Descending	Still	Female	Male	None	Normal	Slow	Very slow
	*M*(SE)	*M*(SE)	*M*(SE)	*M*(SE)	*M*(SE)	*M*(SE)	*M*(SE)	*M*(SE)	*M*(SE)
Liking	36.6 (3.07)	33.6 (3.07)	30.6 (3.07)	38.1 (3.07)	34.1 (3.07)	28.6 (3.07)	36.0 (3.07)	32.5 (3.07)	32.2 (3.07)
Perceived movement	63.5 (3)	59.1 (3)	26.3 (3)	52.2 (3)	51.9 (3)	44.8 (3)	54.7 (3)	49.0 (3)	45.2 (3)
Physical involvement	40.6 (3.52)	38.8 (3.52)	25.2 (3.52)	37.0 (3.52)	38.0 (3.52)	29.6 (3.52)	39.3 (3.52)	34.0 (3.52)	31.4 (3.52)
Emotional involvement	37.6 (3.43)	33.2 (3.43)	34.0 (3.43)	40.3 (3.43)	38.0 (3.43)	26.5 (3.43)	36.8 (3.43)	34.1 (3.43)	33.9 (3.43)
Perceived duration	42.2 (3.27)	42.9 (3.27)	39.4 (3.27)	41.6 (3.27)	41.6 (3.27)	41.3 (3.27)	41.1 (3.27)	41.2 (3.27)	42.2 (3.27)

### Perceived movement

The model explained 73% of the variance in Movement ratings, taking into account the random effects (*R*^2^_m_ = 0.40, *R*^2^_c_ = 0.73). The model revealed a significant main effect of Drone Movement [*χ*^2^_(2)_ = 1117.09, *p* < 0.001], showing that participants perceived more Movement in Ascending than in Descending [*t*_(779)_ = 3.6, SE = 1.22, *p* < 0.01], Ascending more than Still [*t*_(779)_ = 30.6, *p* < 0.001], and Descending more than Still [*t*_(779)_ = 26.97, *p* < 0.001]. A significant main effect for Human Presence was found [*χ*^2^_(2)_ = 47.71, *p* < 0.001], showing that participants perceived more Movement in Female than in None [*t*_(779)_ = 6.08, *p* < 0.001] and in Male with respect to None [*t*_(779)_ = 5.86, *p* < 0.001]. A significant main effect for Image Speed was found [*χ*^2^_(2)_ = 61.67, *p* < 0.001], showing that participants perceived more Movement in Normal than in Slow [*t*_(779)_ = 4.68, *p* < 0.001], in Normal more than Very Slow [*t*_(779)_ = 7.79, *p* < 0.001], and in Slow more than Very Slow [*t*_(779)_ = 3.12, *p* < 0.01].

**Figure 4:**
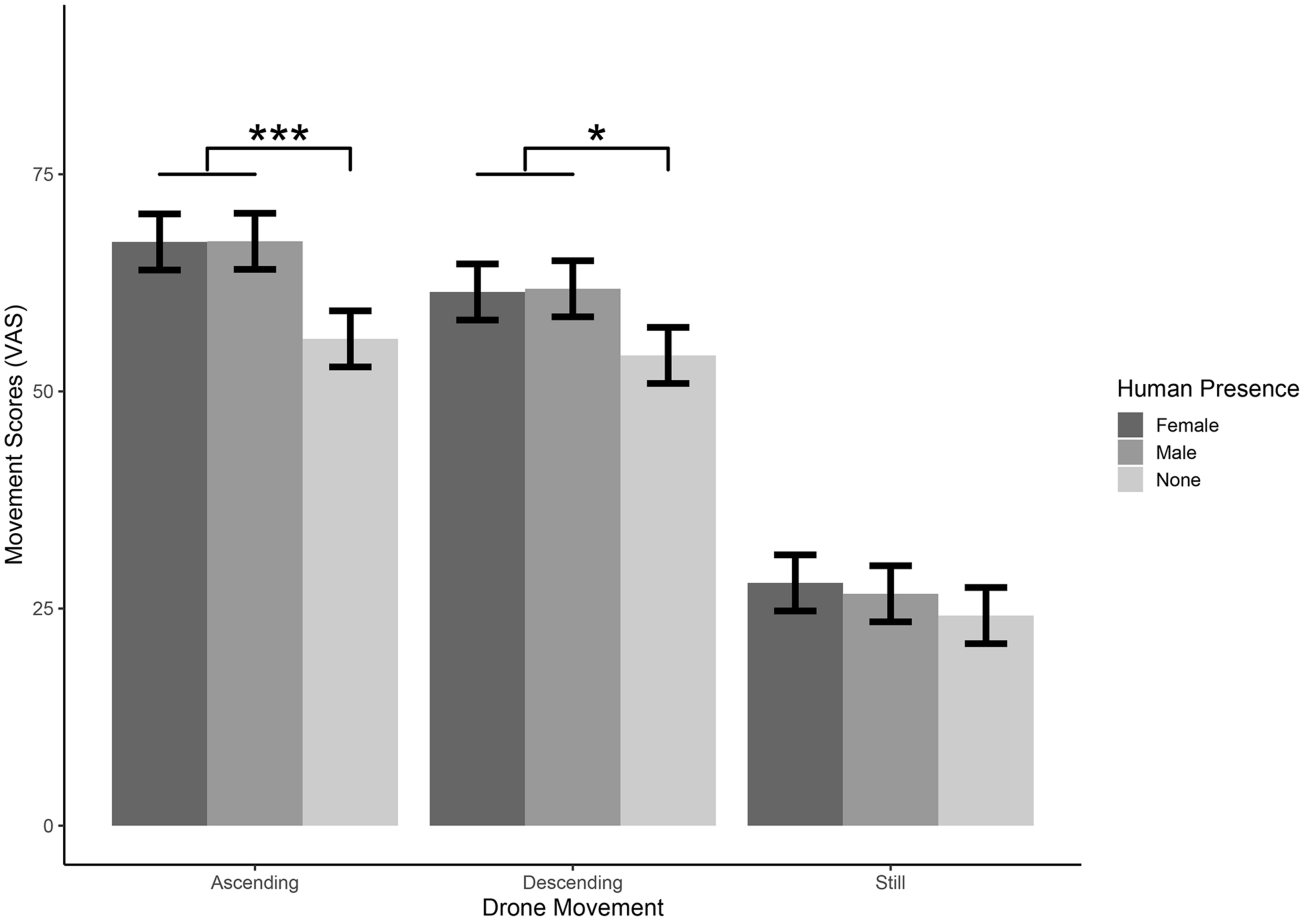
Significant Drone movement*Human presence interaction for the movement question. Please note that differences between Ascending vs. Still and Descending vs. Still are significant for all the three levels of Human Presence (see main text). Error bars represent standard errors of the means-SE. *** = *p* < 0.0001, * = *p* < 0.05.

The model also showed a significant Drone Movement*Human Presence interaction [*χ*^2^_(4)_ = 10.05, *p* < 0.05], showing that for both Ascending and Descending conditions participants perceived more Movement in Female and Male than in None [Ascending Female – Ascending None: *t*_(779)_ = 5.3, *p* < 0.0001; Ascending Male – Ascending None: *t*_(779)_ = 5.33, *p* < 0.001. Descending Female – Descending None: *t*_(779)_ = 3.46, *p* < 0.05; Descending Male – Descending None: *t*_(779)_ = 3.64, *p* < 0.01]. Results also showed that participants perceived more Movement in Ascending and Descending than Still conditions for all the three levels of Human Presence [Ascending Female – Still Female: *t*_(779)_ = 18.63, *p* < 0.001; Descending Female –Still Female: *t*_(779)_ = 15.9, *p* < 0.001. Ascending Male – Still Male: *t*_(779)_ = 19.25, *p* < 0.001; Descending Male – Still Male: *t*_(779)_ = 16.67, *p* < 001. Ascending None – Still None: *t*_(779)_ = 15.07, *p* < 0.001; Descending None – Still None: *t*_(779)_ = 14.17, *p* < 0.001, see Tables 2, 3 and Figure 4].

**Table 3:** Estimated marginal means (M) and standard errors (SE) of significant interaction effects for perceived movement and physical involvement.

	Ascending			Descending			Still		
	Female	Male	None	Female	Male	None	Female	Male	None
	*M*(SE)	*M*(SE)	*M*(SE)	*M*(SE)	*M*(SE)	*M*(SE)	*M*(SE)	*M*(SE)	*M*(SE)
Perceived movement	67.2 (3.23)	67.3 (3.23)	56 (3.23)	61.4 (3.23)	61.8 (3.23)	54.1 (3.23)	27.9 (3.23)	26.7 (3.23)	24.2 (3.23)
Physical involvement	44.2 (3.74)	45.7 (3.74)	32.1 (3.74)	39.9 (3.74)	42.6 (3.74)	33.8 (3.74)	26.9 (3.74)	25.8 (3.74)	22.9 (3.74)

### Physical involvement

The model explained 66.57% of the variance in Physical Involvement ratings, taking into account the random effects (*R*^2^_m_ = 0.12, *R*^2^_c_ = 0.67). The model revealed a significant main effect of Drone Movement [χ^2^_(2)_ = 180.40, *p* < 0.001], showing that participants felt more Physically Involved with Ascending than with Still [*t*_(779)_ = 12.28, *p* < 0.001], and Descending more than Still [*t*_(779)_ = 10.82, *p* < 0.001]. A significant main effect for Human Presence was found [χ^2^_(2)_ = 54.18, *p* < 0.001], showing that participants felt more Physically Involved with Female with respect to None [*t*_(779)_ = 5.91, *p* < 0.001], and more with Male with respect to None [*t*_(779)_ = 6.74, *p* < 0.001]. A significant main effect for Image Speed was found [χ^2^_(2)_ = 41, *p* < 0.001], showing that participants felt more Physically Involved with Normal than with Slow [*t*_(779)_ = 4.2, *p* < 0.001; Normal: *M* = 39.3, SE = 3.52; Slow: *M* = 34.0, SE = 3.52], and with Normal more than with Very Slow [*t*_(780)_ = 6.28, *p* < 0.001; Very Slow: *M* = 31.4, SE = 3.52].

**Figure 5:**
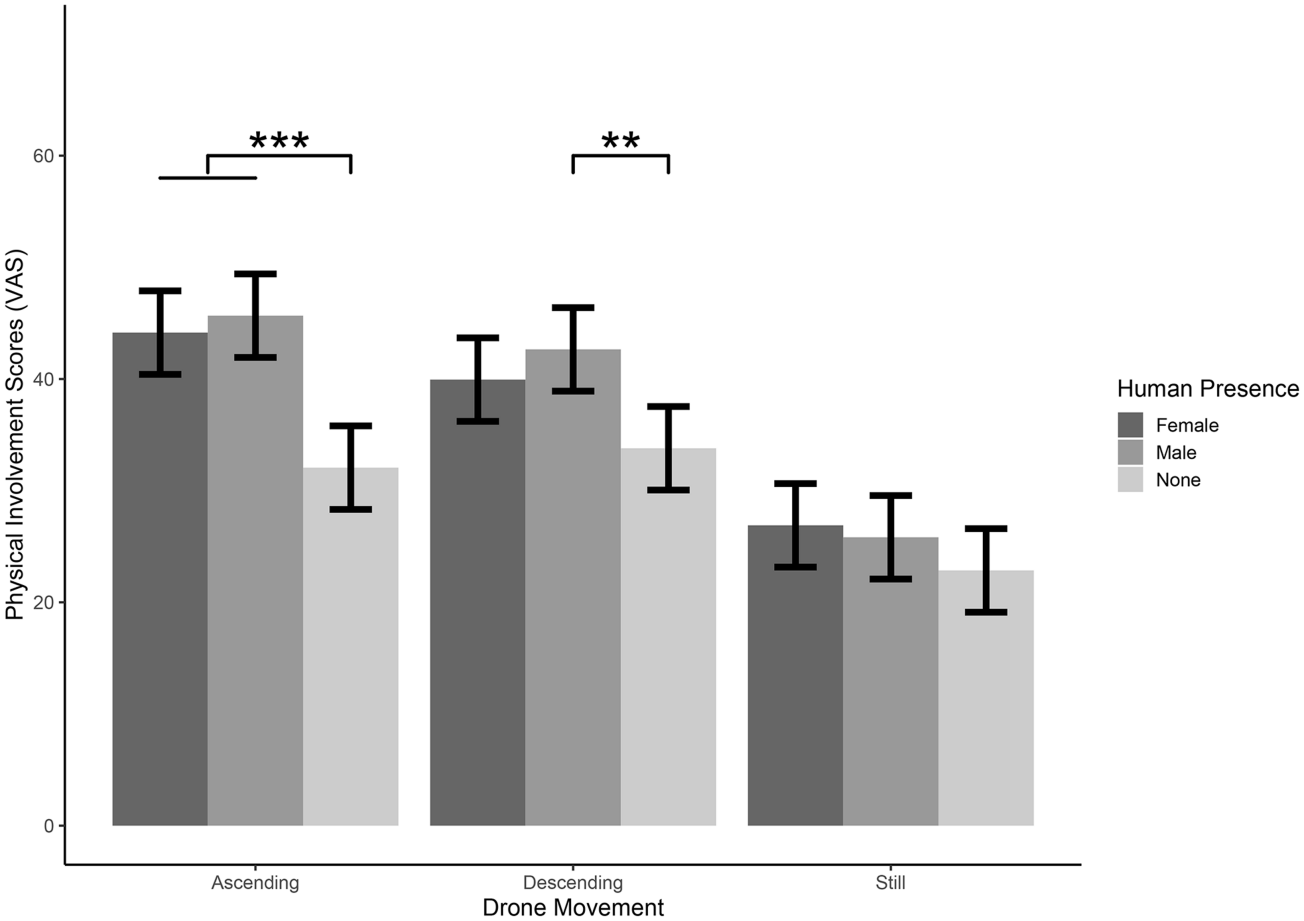
Significant Drone movement*Human preference interaction for physical involvement question. Please note that differences between Ascending vs. Still and Descending vs. Still are significant for all the three levels of Human Presence (see main text). Error bars represent standard errors of the means-SE. * = *p* < 0.05, ** = *p* < 0.001.

The model also showed a significant Drone Movement*Human Presence interaction [*χ*^2^_(4)_ = 13.92, *p* < 0.01], showing that for Ascending condition participants felt more Physically Involved with Female and Male than with None [Ascending Female – Ascending None: *t*_(779)_ = 5.5, *p* < 0.001; Ascending Male – Ascending None: *t*_(779)_ = 6.24, *p* < 0.001]. Participants also felt more Physically Involved with Descending Male than Descending None [*t*_(779)_ = 4.06, *p* < 0.01]. Results also showed that participants perceived felt more Physically Involved with Ascending and Descending than Still conditions for all the three levels of Human Presence [Ascending Female – Still Female: *t*_(779)_ = 7.91, *p* < 0.001; Descending Female – Still Female: *t*_(779)_ = 5.98, *p* < 0.001. Ascending Male – Still Male: *t*_(779)_ = 9.09, *p* < 0.001; Descending Male – Still Male: *t*_(779)_ = 7.71, *p* < 001; Ascending None – Still None: *t*_(779)_ = 4.03, *p* < 0.001; Descending None – Still None: *t*_(779)_ = 4.82, *p* < 0.001, see Tables 2, 3 and Figure 5].

### Emotional involvement

The model explained 63.4% of the variance in Emotional Involvement ratings, taking into account the random effects (*R*^2^_m_ = 0.075, *R*^2^c = 0.63). The model revealed a significant main effect of Drone Movement [*χ*^2^_(2)_ = 13.73, *p* < 0.01], showing that participants found Ascending to be more Emotionally Involving than Descending [*t*_(779)_ = 3.48, *p* < 0.01], and Ascending more than Still [*t*_(779)_ = 2.84, *p* < 0.05]. A significant main effect for Human Presence was found [*χ*^2^_(2)_ = 138.15, *p* < 0.001], showing that participants found Female to be more Emotionally Involving than None [*t*_(779)_ = 10.97, *p* < 0.001], and Male more Emotionally Involving than None [*t*_(779)_ = 9.14; *p* < 0.001]. A significant main effect for Image Speed was found [*χ*^2^_(2)_ = 6.44, *p* < 0.05], but post-hoc tests did not show significant effects. Interactions effects were not significant. See Table 2 for descriptive statistics.

### Perceived duration

The model explained 72.02% of the variance in Perceived Duration ratings, taking into account the random effects (*R*^2^_m_ = 0.01, *R*^2^_c_ = 0.72). The model revealed a significant main effect of Drone Movement [*χ*^2^_(2)_ = 15.51, *p* < 0.001], showing that participants found Ascending to have a longer perceived Duration than Still [*t*_(779)_ = 3, *p* < 0.01], and Descending more than Still [*t*_(779)_ = 3.51, *p* < 0.001], with no significant difference between Ascending and Descending. No significant main effects were found for Human Presence and Image Speed, nor for interactions effects. See Table 2 for descriptive statistics.

### Correlations

**Figure 6:**
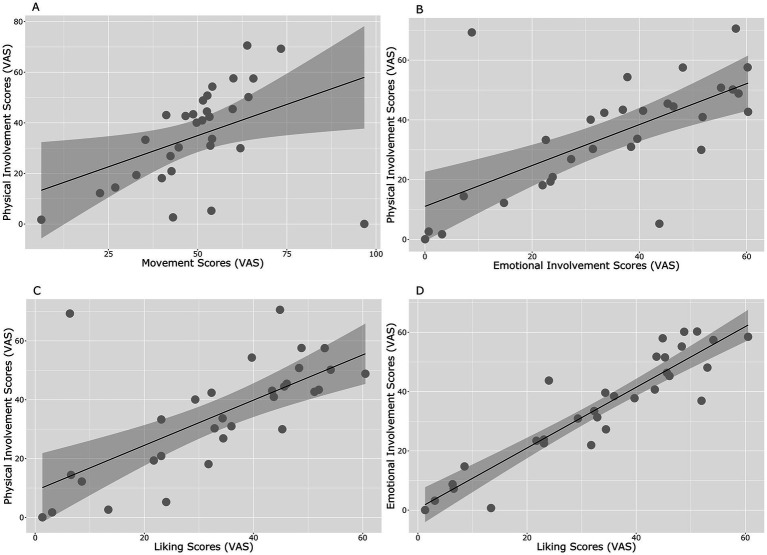
Significant correlations. Physical Involvement*Perceived Movement **(A)**, Physical Involvement*Emotional Involvement **(B)**, Physical Involvement*Liking **(C)**, and Emotional Involvement*Liking **(D)**.

Results of the Spearman ranks correlations (see Figure 6) indicate that after Bonferroni correction (*p* = 0.05/10 = 0.005), four positive correlations resulted significant: Physical Involvement*Perceived Movement (Rho = 0.56, *p* = 0.001; Figure 6A), Physical Involvement*Emotional Involvement (Rho = 0.68, *p* < 0.0001; Figure 6B), Physical Involvement*Liking (Rho = 0.71, *p* < 0.001; Figure 6C), and Emotional Involvement*Liking (*R* = 0.89, *p* < 0.001; Figure 6D). No other significant correlations were found.

## Discussion

In the present study, we investigated the impact of Drone Movement, Human Presence and Image Speed on participants’ ratings of Liking, Perceived Movement, Physical Involvement, Emotional Involvement, and Perceived Duration. In order to increase the ecological validity of the study, we collaborated with a professional filmmaker and a drone pilot to create 81 novel and carefully controlled video stimuli modeled after the staircase scene in *The Cranes Are Flying* (Kalatozov, 1957). This scene was chosen in particular due to its expression of cinematic affect through the motif of “rising” in the embodied aesthetics of the actor and the camera (Kolesnikov, 2022). Its use as a template for stimuli creation enabled the manipulation and control for multiples variables of interest.

Firstly, for the dependent variable Liking, results demonstrate that there are significant main effects for Drone Movement, Human Presence and Image Speed. It was shown that Ascending received significantly higher ratings for Liking than Descending and Still, and Descending more than Still. It was also shown that Female received significantly higher ratings for Liking with respect to Male, which suggests that Female conditions are found to be more pleasing in terms of appearance or movement fluidity, and Female and Male have significantly higher ratings with respect to None, suggesting that the appeal of the stimuli is enhanced with an actor, whose presence attributes meaning to the context of the video (i.e., a staircase).

Results for Perceived Movement measure suggest that there are significant main effects for Drone Movement, Human Presence and Image Speed. It was demonstrated that, in line with our hypothesis, Ascending and Descending received significantly higher ratings for Movement with respect to Still and, notably, that Ascending was perceived to evoke more Movement than Descending. These results are in line with prior neuroimaging studies on sensorimotor engagement during the observation of human and camera movement (e.g., Buccino et al., 2001; Aziz-Zadeh et al., 2006; Calvo-Merino et al., 2005, 2006; Orgs et al., 2008; Aglioti et al., 2008; Abreu et al., 2012; Heimann et al., 2014, 2019), and provide further evidence in support of embodied simulation theory. Furthermore, a distinction has been demonstrated in the perception of Ascending with respect to Descending, suggesting that Ascending movement, embodied in the act of running up the stairs against the force of gravity, requires greater effort (i.e., exertion), and this greater exertion modulates the perception of movement in participants. Neuroimaging studies are needed in order to investigate differences in sensorimotor engagement between Ascending and Descending drone/actor movement, to see whether this is also supported by differences at the neural level. It was also found that Female and Male Human Presence were rated as evoking significantly more Perceived Movement with respect to None (with no significant difference between Female and Male), which is in line with the “doubly” embodied nature of Female and Male conditions. That is to say, whereas the None condition has one vector of movement, i.e., the drone, Female and Male conditions have two, i.e., the drone and the human body. Results also show that Normal conditions were perceived as evoking more Movement than Slow and Very Slow, and Slow more than Very Slow, indicating greater perceived embodiment for “natural” movement (i.e., original image speed) than for “slow motion” movement. Participants rated Perceived Movement precisely in terms of visualized apparent motion in a given time frame, rather than in terms of perceived effort. Unlike the results related to Drone Movement (i.e., ascent), which seem to imply a motor resonance linked to physical exertion, the results related to Image Speed appear to be linked to the visual ecology of the observed movement. Specifically, when Image Speed decelerated, the speed of human movement also decelerated, reducing the total quantity of perceived motion in the 10-s stimuli. One possible interpretation is that the slow motion falls outside the observers’ motor repertoire, attenuating their motor resonance with bodies moving in slow motion.

For Physical Involvement, results demonstrate significant main effects for Drone Movement, Human Presence and Image Speed. Like for the Perceived Movement measure, it was found that Ascending and Descending were perceived as evoking more Physical Involvement than Still. It was also found that None clips were rated as significantly less Physically Involving than Female and Male ones, again supporting embodied simulation theory, which suggests that we resonate more with conspecifics and familiar motor repertoires. Although the absence of others in the video image may increase the impression of “immersion” in the scene and, as a result, identification with the drone and simulation of its rotatory trajectory in space, participants found actor presence to be more involving on a motor level. In fact, results from the Drone Movement*Human Presence interaction effect demonstrate that even in the absence of an actor, participants felt more Physically Involved when the drone was moving (i.e., Ascending/Descending). This supports the research of Heimann and co-authors (2014; 2019), which demonstrates that video clips filmed with a Steadicam are perceived as more engaging, natural, and simulatory of the approach of a human observer with respect to zoom, dolly or still shots. Finally, contrary to our hypothesis and similarly to the Movement results, participants felt more physically involved by the Normal conditions than by the Slow and Very Slow ones. It is therefore possible that the spectators perceive the lightness of their point of view, i.e., the drone “floating” in the air, more than the sense of effort observed in the actor. Future studies oriented toward a phenomenological investigation are needed to further explore this possibility and its implications. Results for Emotional Involvement revealed significant main effects for Drone Movement, Human Presence and Image Speed. Participants rated Ascending as more Emotionally Involving than Still and, notably, Descending, suggesting that the stronger emotional angagement of Ascending movement may stem from greater perceived exertion and thus stronger sensorimotor engagement. While both Ascending and Descending clips involve goal-oriented movements – “ascent” appears more emotionally impactful than “descent,” possibly due to its association with the motif of “rising”/“upness,” which carries a positive embodied meaning compared to “staying”/“standing” or “downward”/“falling.” Future studies could explore whether Ascent is linked to “positive” valence and Descent to “negative” valence. This idea aligns with Conceptual Metaphor Theory (CMT; Lakoff and Johnson, 1980), a core framework within embodied cognition that posits abstract cognitive domains are rooted through metaphorical mappings in concrete, physical experiences, and thus in sensorimotor experiences. In this case, the relevant metaphor is “GOOD IS UP/BAD IS DOWN,” which suggests that upward movement is typically associated with positive valence, while downward movement tends to carry negative connotations. Notably, this association is not arbitrary but emerges from recurrent bodily experiences. Nonetheless, the greater emotional impact of Ascending movement supports Kalatozov’s artistic choice to associate the protagonist’s joy with the motif of physical rising, epitomized by the staircase, and reinforces the broader emotional metaphor of joy as ascent. It was also found that Female and Male are more Emotionally Involving than None, indicating that an actor’s presence adds meaning to the scene and enhances viewers’ emotional identification.

Finally, for the dependent variable Perceived Duration, results demonstrate a significant main effect for Drone Movement. Ascending and Descending conditions were perceived as having a longer Duration than Still (even though all stimuli have a duration of 10 s). Such a result is coherent with previous evidence showing that people are likely to perceive the duration of moving stimuli as longer than that of stationary stimuli, even when their physical duration is identical (e.g., Brown, 1995; Kanai et al., 2006; Eagleman, 2008; Balzarotti et al., 2021). Contrary to our hypothesis, no significant differences were found between conditions for perceived Duration for the main effect Image Speed. Rather than a distortion in the perception of subjective time in slow motion conditions, participants perceived stimuli durations in terms of their actual running time.

In sum, these results showed that ascending movements elicited the highest levels of perceived movement, aesthetic appreciation, and emotional engagement, outperforming descending and still conditions. These results may be explained by the stronger sense of effort and exertion associated with ascending movements, which intrinsically go against gravity, evoking potentially stronger embodied responses in the viewer. This is also closely related to the concept of “animacy” in the context of object kinematics: evidence suggests a tendency to perceive the upward movement of an object, even an inanimate one, as more animated than movement in the opposite direction (e.g., Szego and Rutherford, 2008; Nguyen and van Buren, 2023). For a review (see Parovel, 2023).

While one might consider that the rotational movement of the drone around its vertical axis may also modulate these responses, the significant differences between ascending and descending movements effectively disentangle the contribution of the two types of motion (vertical and rotational), as the rotational component is equally present in both conditions. Therefore, any differences can be attributed solely to vertical motion (i.e., upward or downward motion along the vertical axis). Additionally, the presence of a human significantly enhanced ratings across all metrics compared to videos without human figures, with the female actress receiving higher aesthetic ratings. The significant positive correlation between Physical Involvement and Movement indicates that participants who felt more physically engaged with drone and human movements also experienced a stronger sense of embodiment. This finding aligns with research suggesting that motor resonance and embodiment are fundamental to the perception of movement (e.g., Gallese et al., 2004). Similarly, the strong correlation between Physical and Emotional Involvement underscores the deep connection between bodily engagement and emotional experience. In particular, for a phenomenological exploration of the possible relations between spectators’ emotional involvement and actors’ bodily actions performed on screen see (Michotte, 1953). The relationship between Physical Involvement and Liking suggests that greater physical engagement with drone and human movements enhances appreciation and enjoyment. This observation resonates with studies on sensorimotor coupling, which emphasize how active physical engagement can heighten aesthetic experiences and preferences (e.g., Leder et al., 2012; Ardizzi et al., 2020). Additionally, the strong positive correlation between Emotional Involvement and Liking highlights the essential role of affect in shaping aesthetic preferences. These findings demonstrate, for the first time, that observing footage filmed with a drone can elicit interconnected dynamics of physical and emotional engagement, as well as influence aesthetic preferences. They also support theories of embodiment in movement perception and point to practical applications of drone-filmed footage in audiovisual media such as cinema, virtual reality, and game design. Understanding the bodily basis of viewer engagement is indeed critical for enhancing immersive storytelling. Furthermore, the strength of the emotional-liking correlation suggests that fostering emotional resonance is key to enhancing audience engagement in interactive settings.

A central aim of this study was to recreate the iconic staircase scene from The Cranes Are Flying, selected for its expressive integration of emotional content and complex camera movement. In the original film, this sequence was achieved through an elaborate elevator crane system—custom-built with a cradle for the cinematographer and circular tracks to enable a fluid lateral ascent (see Kolesnikov, 2022). Reproducing such a setup indoors would have required considerable time, resources, and structural modifications. Instead, we employed a drone, which allowed us to replicate the scene’s intricate circular motion with minimal setup. The drone’s agility and remote operation made it especially well-suited for navigating the confined vertical space of the stairwell, offering smooth, continuous motion without the logistical demands of cranes or body-mounted rigs. In this context, the drone provided an efficient and cost-effective solution for capturing dynamic, multi-directional movement indoors—delivering camera trajectories that would otherwise necessitate a combination of pulley systems, rigging, and traditional camera mounts. In conclusion, in the present study, we demonstrate how drone-filmed video clips, with and without human presence, impact aesthetic evaluation, perceived movement, physical involvement, emotional involvement, and perceived duration. Considering also our previous musical study (Kolesnikov et al., 2023), we thus present evidence for an “ascension-exertion-effect” not only through musical movement but also through visual movement, both of which support the theory of embodied simulation and the frameworks of embodied cinema. In the future, it would be valuable to (i) combine visual and auditory modalities to enhance our understanding of the filmic experience; (ii) examine how drone movement, with or without human bodily movement, impacts cortical sensorimotor activation in the brain; and (iii) explore participants’ experiences from a phenomenological perspective, such as feelings of lightness and suspension, as well as potential effects related to the drone’s rotational movement.

## Data Availability

The raw data supporting the conclusions of this article will be made available by the authors, without undue reservation.
