# Supplementary material for: Simulating drone and bodily movements: a behavioral study
Source: Front Psychol. 2025 Apr 25;16:1559756. doi: 10.3389/fpsyg.2025.1559756 (PMC12062006; doi:10.3389/fpsyg.2025.1559756)
Supplement: Supplementary file 1 [file Data_Sheet_1.pdf]

Supplementary Materials

Simulating drone and bodily movements: a behavioral study

Anna Kolesnikov[1,2]*, Marta Calbi[2,3]*, Martina Montalti[2,4], Nunzio Langiulli[2], Michele Guerra[5], Vittorio Gallese[2,6], Maria Alessandra Umiltà[3,6]

[1] Department of Art History, Film and Audiovisual Media Studies, Université de Montréal, Montreal, Canada
[2] Department of Medicine and Surgery, Unit of Neuroscience, University of Parma, Parma, Italy

[3] Department of Food and Drug, University of Parma, Parma, Italy

[4]Department of Ancient and Modern Civilizations, Polo universitario "Annunziata", University of Messina, Messina, Italy

[5] Department of Humanities, Social Sciences, and Cultural Industries, University of Parma, Parma, Italy
[6] Italian Academy for Advanced Studies in America, Columbia University, New York, USA

*These authors contributed equally to this work and share first authorship.

Stimuli

Motion analysis and results

The main objective of motion analysis was to verify that there were no significant differences between Ascending and Descending conditions, and between Female and Male conditions. Motion analysis was carried out using PIVlab, a free toolbox and app for MATLAB. PIVlab is an open-source particle image velocimetry (PIV) software that both calculates the velocity distribution within image pairs and derives, displays and exports multiple parameters of the flow pattern. PIVlab uses a coordinate system in which the standard coordinate system of image data has its origin at the top left, where velocities are measured in both horizontal (x) and vertical (y) directions; positive x is top-to-bottom, and positive y is left-to-right. PIVlab's default correlation algorithm is FFT window deformation - which employs direct Fast Fourier Transform correlation with multiple passes and deforming windows. Data were analyzed by selecting and configuring "interrogation areas" to reduce the signal-to-noise ratio and increase cross correlation robustness. The analysis began with an interrogation area of 128 pixels, then decreased to 64 pixels and finally to 32 pixels. Vector magnitude for each frame (250 frames in total) were exported as MAT files for each of the 27 video files. The mean velocity magnitude per video file was then extracted in R Studio, and used for a two-tailed t-test that compared Ascending vs. Descending and Female vs. Male conditions.

Results of the t-Test indicate no significant differences between Ascending and Descending (p = .13; Ascending: M = 27.39, SD = 1.28; Descending: M = 26.25, SD = 1.71), and between Female and Male (p = .09; Male: M = 18.38, SD = 13.16; Female: M = 17.62, SD = 12.69) conditions.

Luminance analysis and results

The mean luminance levels of videos were extracted using a custom MATLAB script. For each video the script loaded the file and determined its frame count, resolution, and dimensions. Then, the script iterated through all frames of each video, converting each frame to grayscale and calculating the mean pixel intensity for that frame. To investigate whether mean luminance levels differed across Drone Movement, Human Presence and Image Speed, a linear mixed effect analysis was carried out. Mean luminance levels were entered as dependent variables, (Drone Movement (3 levels: Ascending, Descending, Still), Human Presence (3 levels: Female, Male, None) and Image Speed (3 levels: Normal, Slow, Very Slow) as independent fixed variables, and videos and frames intercepts as random effects. Tukey's test was used for post-hoc comparisons among means.

The model explained 74.4% of the variance, taking into account the random effects ($R^2_m = 0.11$, $R^2_c = 0.74$). The model revealed a significant main effect of Drone Movement ($\chi^2_{(2)} = 51.16$, $p < .001$), showing higher mean luminance values for Still than Ascending ($z_{(inf)} = -6.86$ $p < .001$; Ascending: M = 82.5, SE = 2.76; Still: M = 109.3, SE = 2.76), and Descending ($z_{(inf)} = -5.17$, $p < .001$; Descending: M = 89.1, SE = 2.76). The model also showed a significant Drone Movement*Human Presence interaction ($\chi^2_{(4)} = 9.95$, $p < .05$, Table S2), showing that mean luminance levels were significantly higher for Still than Ascending and Descending videos for both Female and Male actors while there were no differences for None condition (Female Ascending: M = 77.5, SE = 5.11; Female Descending: M = 81.9, SE = 5.11; Female Still = 112.3; 5.11; Male Ascending: M = 83.9, SE = 5.11; Male Descending: M = 88.3, SE = 5.11; Male Still = 114.9, 5.11).

	z	*p*
**Female**		
Ascending vs. Still	-5.14	<.001
Descending vs. Still	-4.5	<.001
**Male**		
Ascending vs. Still	-4.56	<.001
Descending vs. Still	-3.93	<.001

Table S1. Luminance analysis post hoc comparisons.

Control analysis on participants' gender

A t-test was conducted to compare the VAS ratings given by male and female participants for each question. No significant differences were found between Male and Female participants.

Liking: $t_{(27.246)} = 1.1993$, $p > .05$, (95% CIs: -7.51, 28.68).

Movement: $t_{(27.902)} = -0.24843$, $p > .05$, (95% CIs: -14.72,11.54).

Physical Involvement: $t_{(28.881)} = 1.6305$, $p > 0.5$, (95% CIs: -4.17, 36.95).

Emotional Involvement: $t_{(28.434)} = 1.081$, $p > 0.5$, (95% CIs: -10.21, 33.05).

Duration: $t_{(22.735)} = -0.76564$, $p > .05$, (95% CIs: -22.82, 10.49).
